# Prevalence of risky alcohol use behavior and associated factors in pregnant antenatal care attendees in Debre Berhan, Ethiopia, 2018

**DOI:** 10.1186/s12888-019-2225-1

**Published:** 2019-08-13

**Authors:** Abate Dargie Wubetu, Surafel Habte, Kefyalew Dagne

**Affiliations:** 1 Department of Psychiatry, College of Health Science and Medicine, Debre Berhan University, P.O. Box 445, Debre Berhan, Ethiopia; 2 Debre Berhan Public Health Institution, Debre Birhan, Ethiopia

**Keywords:** Alcohol, Risky use, Pregnancy

## Abstract

**Background:**

National Institute of alcohol and alcoholism define Problem/risky use of alcohol as drinking in a way that can negatively impact our health and life, but the body is not physically dependent on the substance. Risky alcohol use behavior is harmful to both the fetus and the mother such as maternal alcohol intoxication and alcohol use disorder, abortion, stillbirth, low birth weight, and prematurity. This study is aimed to assess the prevalence of risky alcohol use for both the mother and the fetus during pregnancy among mothers who have used alcohol at least once in the current pregnancy.

**Methods:**

Institution based cross-sectional study was conducted among a total of 380 mothers who have used alcohol at least once during the current pregnancy. The participants were selected using a systematic random sampling technique. Both Bivariable and Multivariable Binary Logistic Regression models were done to identify associated factors. Odds Ratios with their 95% Confidence Interval was computed and variables with a *p*-value < 0.05 during multivariable analysis were considered significantly associated factors.

**Results:**

A total of 380 mothers who used alcohol at least once (any amount) in the current pregnancy were included in the study and further interviewed for risky alcohol use behavior with a response rate of 100%. The overall prevalence of risky alcohol use behavior was 16.1% (95% CI = 12.1, 19.7). Having poor social support, having moderate to severe depression and anxiety, having diagnosed family history of mental illness, having a history of abortion were important factors which significantly associated with risky alcohol use behavior.

**Conclusion:**

Significant proportions of pregnant mothers were risky alcohol drinkers. It will be better if screening of any amount of alcohol use during pregnancy and providing health education (about the risk of alcohol use) for all pregnant women who attend antenatal care follow up.

## Introduction

National Institute of alcohol and alcoholism define Problem/risky use of alcohol as drinking in a way that can negatively impact our health and life, but the body is not physically dependent on the substance [1].

World health organization report on alcohol drinking recommended that there is no safe amount and type of alcohol drinking during pregnancy. The recommendation also warns alcohol is the most common teratogen and harmful substance and no safe time to drink during pregnancy [2].

Excessive alcohol use during pregnancy can result in major body organs birth defect, developmental problems and damage of multiple brain structures; resulting in lifelong disabilities, multiple health and social problems for both the mother and child. Among health impacts of alcohol use during pregnancy miscarriage, stillbirth, low birth weight, and prematurity were seen commonly [3].

The effect of risky alcohol use on behavior, health, and society are the main challenges across the world [4]. Alcohol is the most common teratogen and harmful substance if consumed during pregnancy; it can cause a problem for pregnancy and puts the mother at risk [5].

Maternal alcohol use during pregnancy increases the risk and occurrence of preterm delivery and spontaneous abortion. Its significant risk of mental, physical and psychological harm to the fetus is well known; called fetal alcohol spectrum disorders (FASD) and fetal alcohol syndrome (FAS) [6–8]. According to the report of the World Health Organization (WHO) prenatal alcohol use or exposure determinant for intellectual and developmental disabilities [9]. In addition, neurocognitive deficits manifested in language, motor, and cognitive functions are verified as the impact of alcohol use during pregnancy. Moreover, multiple body organ problems i.e. cardiac, skeletal, renal, ocular and auditory deficits have been proved as the effect of alcoholic beverages drinking during pregnancy [10].

Alcohol use during pregnancy often occurs in concordance with other predictors; such as maternal smoking and family of alcohol abuse. So, sometimes it is difficult to attribute the effects to fetal alcohol exposure or to the characteristics of the mother [11].

The excessive use of alcohol in the early weeks of pregnancy may lead to spontaneous abortion, and its consumption between the third and eighth week of pregnancy could increase the risk of physical deformities in the fetus. The effect of alcohol on the newborn is manifested through the Fetal Alcohol Syndrome (FAS); 33% of children born from mothers who used more than 150 g of ethanol per day will develop fetal alcohol syndrome. In addition, children of women who consumed alcohol moderately may present agitation, breast suction deficiency, sweating, and changes in sleep patterns illustrates the condition of abstinence syndrome [12].

According to the Ethiopia Demographic and Health Survey (EDHS) report in 2011, 45% of women reported drinking alcohol at least once in a lifetime; this proportion increases with age, and it is higher among urban areas than in rural areas. In addition to the above, the majority of the community had habits to prepare alcoholic beverages at their home in every ceremony and holidays. Homemade alcoholic beverages are common in the study area and used by anyone without the restriction of gender, age and pregnancy status by ignoring its unfavorable consequence [13].

Any amount of alcohol use during pregnancy at least once during pregnancy is well studied in Ethiopia. Even if drinking of alcoholic beverages in any amount is teratogen for the growing fetus but this study aims to determine proportions of mothers with risky alcohol drinking behavior (both for the mother and the fetus) during pregnancy; as it is important for policymakers and health professional to design health education strategies for mothers who had ANC follow up at health clinics.

### Specific objectives

- To determine the prevalence of risky alcohol use behavior in pregnant antenatal care attendees
- To identify factors associated with risky alcohol use behavior in pregnant antenatal care attendees.

### Hypothesis

- Ho_1_: there is no risky alcohol use behavior among pregnant mothers.
- Ho_2_: all pregnant mothers with different characteristics are equally experienced risky alcohol use behavior.

## Methods

### Study area

Debre Berhan town is located in North Shoa Administrative Zone, Amhara National Regional State, Ethiopia. It is found at 130 km North of Addis Ababa; the capital city of Ethiopia and 695 km from Bahir-Dar; the capital of Amhara regional state. The town is founded by Emperor Zara Yakob on 7 March 1456. The town has 9 administrative Kebele. In the town, there is one public referral hospital and one private general hospital, 3 health centers, 9 health posts, 5 private specialty clinics, which render health services for the community. These public health institutions expected to serve 2.8 million peoples per year and has Adult and pediatric outpatient, Immunization room, Emergency room, labor and delivery room, laboratory and antenatal care unit rooms. The above health institutions have the same national standard and private specialty clinics were not included in the study.

### Study design and period

Institutional based cross-sectional study was conducted from April–May 2018.

### Population

#### Source population

All pregnant women who have used alcohol at least once in the current pregnancy and living in Debre Berhan town and surrounding catchment areas.

#### Study population

All pregnant women who have used alcohol at least once in the current pregnancy and had ANC follow up in public health institutions in Debre Berhan town.

### Eligibility

#### Inclusion criteria

All pregnant women who have used alcohol at least once in the current pregnancy; had ANC follow-up, and who were available during the study period.

#### Exclusion criteria

Pregnant women who were severely ill to the extent of interviewing were excluded.

### Sample size calculation and sampling procedures

The sample size was calculated using the general formula for single population proportion and by considering 5% margin of error, Z = 1.96 (95% confidence level) and proportion = 34% of respondents consumed alcohol during pregnancy in a similar study [14]. By adding a 10% non-response rate the final sample size of the study became (*n* = 380). The Preliminary assessment study was done among all pregnant women who had antenatal care follow up at all public health institutions. Based on the assessment 685 pregnant mothers’ use any amount of alcoholic beverage at least once during the current pregnancy were included in the study. Based on the baseline information, pregnant mothers (current users) were further interviewed for risky alcohol use behavior by using systematic random sampling technique (k = 2).

### Study variables

#### Dependent variables

Risky Alcohol Use behavior (yes /No).

#### Independent variables

Socio-demographic characteristics: (age, religion, ethnicity, marital status, educational, occupation, and monthly income).

Obstetric factors: - (parity, gestational age, number of children, pregnancy plan status, previous history of abortion/stillbirth, complication during pregnancy).

Psychosocial Factors**:** -(social support, Violence (sexual/physical), stressful life events).

Clinical Factors: - chronic medical illness, history of mental illness, family history of mental illness, depression, and anxiety.

### Operational definitions

Risk drinking/ Problem drinking: pregnant Mothers who scored 2 or more on “CAGE” alcohol use screening tool (CAGE: C- Cut down, A- annoyed, G-guilty feeling, and E-Eye opener).

Depression and anxiety: - According to patient’s health questionnaire (PHQ-4), individuals for depression and anxiety were rated as normal (0–2), mild [3–5], moderate [6–8], and severe [9–12]. Total score ≥ 3 for the first 2 questions suggests anxiety. And total score ≥ 3 for the last 2 questions suggests depression.

Poor support:-Mothers who score 3–8 on the OSLO-3 social support scale during pregnancy.

Moderate support:-Mothers who score 9–11 on OSLO-3 social support scale during pregnancy.

Strong support:-Mothers who score 11–14 on the OSLO-3 social support scale during pregnancy.

Violence: - According to Abuse Assessment Screen (AAS) for use in Pregnancy, at least a response of one “yes” for physical or sexual abuse from three questions.

Stressful Life event: according to the list of threatening events questionnaire (LTE-Q), individuals who reported no “yes” for all items considered as no trauma, one “yes” as exposed to single trauma and two or more “yes” considered as exposed to multiple trauma.

### Data quality control, data collection procedure, and tools

The questionnaires were translated into the local language (Amharic) and back to English; and was checked for consistency. Study subjects were informed on questions included in the questionnaires, purpose of the study, importance of privacy, and confidentiality issues. Before conducting the main study, a pretest was carried out in another health institution before two weeks of the main data collection period on 38 (10%) individuals who were attending antenatal care unit and the pretest data was not included in the analysis.

Data were collected by using pretested semi-structured data-collection tools for some associated factors. Standardized tools were used to assess the outcome variable and some explanatory variables. All the data’s required were quantitative and was collected by interviewer-administered questionnaire. The CAGE questionnaire was used to assess risky alcohol use behavior during pregnancy. The patient’s health questionnaire (PHQ-4) was used to assess the level of anxiety and depression, Oslo-3 to assess social support level, Abuse Assessment Screen (AAS) to assess the status of physical emotional and sexual abuse status and list of threatening events questionnaire (LTE-Q) to assess pregnant women level of exposure to life-threatening events.

### Data processing and analysis

Data was entered into Epi-data version 3.1 statistical software after coding and checking of the questionnaires. The entered data were exported to SPSS version 20 for analysis. Then data were categorized and sorted to facilitate its analysis. Descriptive statistics were used to describe the frequency and percentages of the respondents by socio-demographic characteristics, obstetric factors, Psychosocial Factors, and Clinical Factors. Bivariate and multivariate Binary logistic regression models were used to identify factors associated with risky alcohol use behavior.

The descriptive results were presented in text and tables as based on the types of data. All explanatory variables which were associated with the outcome variable during bivariate analysis with a *p*-value of 0.2 or less were exported to multivariate analysis. The crude and adjusted odds ratio together with their 95% confidence intervals were computed. During multivariate binary logistic regression, *P*-value < 0.05 was considered to declare as statistically significant associated factors. Assumption tests of logistic regression were managed. The value of VIF (variance inflation factor) was above 1 and below 5 for multicollinear variables. The Model goodness of fit was done by using the Hosmer Lemeshow test and its *p*-value was 0.54.

### Ethical consideration

Ethical clearance was obtained from the Institutional Health Research Review Committee (Ref. No. IHRERCB-569/2018) of the College of Health and Medicine, Debre Berhan University. Official permission was taken from each health institution administrators and verbal consent was taken from the study participants. All pregnant women who use alcohol were informed about the effect of alcohol.

## Results

### Socio-demographic characteristics

A total of 380 respondents participated in the study, yielding a response rate of 100%. A total of 380 pregnant women were interviewed in the study; of them, almost 82% (*n* = 310) were found between the age of 25–45 years old (mean age = 29.6 ± 5.7 years). The majority, 275(72.4%) were married and 51(13.4%) were single. Regarding occupation, the higher proportion of the study subjects, 133(35%) were a private employee. As it would be expected based on population values of the study area, a vast majority 244 (64%) of the respondents’ were Amhara in ethnicity, and 262 (66%) of the study subjects were Orthodox by religion (see Table 1).

**Table 1:** Socio-demographic characteristics of study mothers on antenatal care follow-up at public health institution in Debre Berhan, April 2018(*n* = 380)

Variables		Frequency (%) (n = 380)
Age	18–24 (youth)	70 (18.4)
	25–45(young adulthood)	310 (81.6)
Religion	Orthodox	262 (65.9)
	Muslim	98 (25.8)
	Protestant	20 (5.3)
Marital status	Single	51 (13.4)
	Married	275 (72.4)
	Divorced	38 (10)
	Widowed	16 (4.2)
Monthly income (quartile)	≤ 1200	97 (25.5)
	1201–2500	102 (26.8)
	> 2500	181 (47.6)
Family size	1–2	103 (27.1)
	3–4	204 (53.7)
	≥ 5	73 (19.2)
Ethnicity	Amhara	244 (64.2)
	Oromo	93 (24.5)
	Tigre	25 (6.6)
	Gurage	18 (4.7)
Educational level	Not attend modern education	99 (26.1)
	Primary school	104 (27.4)
	Secondary school	67 (17.6)
	Diploma	78 (20.5)
	Degree and above	32 (8.4)
Occupation	Government employee	72 (18.9)
	Unemployed	80 (21.1)
	private employee	133 (35)
	Housewife	86 (22.6)
	Student	(2.4)

### Obstetric factors

From all pregnant women, 271(71%) of mothers reported that the intention of the current pregnancy was wanted and 251(66%) of the pregnancies were planned. Almost half, 186 (48.9%) of the mothers were in the third trimester of pregnancy. Twenty-eight percent (*n* = 108) of the study subjects had no child. History of abortion was experienced by 68 (17.9%) of respondents. Though, 17.6, 46.1, 25.3 and 11.1% of participants reported to have ANC follow up in first, second, third and fourth visit respectively; 233(61%) of them were not informed about the risks of drinking alcohol by their health care providers (see Table 2).

**Table 2:** Obstetric history of study mothers on antenatal care follow-up at public health institution in Debre Berhan, April 2018 (*n* = 380)

Variables		Frequency (%) (n = 380)
Pregnancy intention	Planned	251 (66.1)
	Unplanned	129 (33.9)
Current pregnancy	Wanted	271 (71.3)
	Unwanted	109 (28.7)
Gravidity	Prim gravida	50 (13.2)
	Multigravida	330 (86.8)
Parity	No child yet	108 (28.4)
	One child	105 (27.6)
	Two child	125 (32.9)
	≥ 3 child	42 (11.1)
Weeks of pregnancy	First trimester	42 (11.1)
	Second trimester	152 (40)
	Third trimester	186 (48.9)
ANC visit	First visit	67 (17.6)
	Second visit	175 (46.1)
	Third visit	96 (25.3)
	Fourth visit	42 (11.1)
Previous history of still death	Yes	38 (10)
	No	342 (90)
Previous history of abortion	Yes	68 (17.9)
	No	312 (82.1)
Previous neonatal death	Yes	25 (6.6)
	No	355 (93.4)
Previous pregnancy complication	Yes	68 (17.9)
	No	312 (82.1)
Current pregnancy complication	Yes	47 (12.4)
	No	333 (87.6)
Informed about alcohol drinking on ANC	Yes	147 (38.7)
	No	233 (61.3)

### Clinical factors

Of 380 respondents, 23 (6.1%) had known psychiatric illness and 45(11.8%) of the respondent’s family had known mental health problem. Self-reported diagnosed medical illness (pregnancy-related or previously diagnosed) was found among 54(14.2%) of pregnant women.

### Psychosocial factors

Study subjects social support status was screened by using the OSLO-3 social support scale. Among all study subjects, 171 (45%) had moderate social support and almost half of the study participants had moderate husband support during their pregnancy. Almost 56% (*n* = 193) of the study subjects didn’t experience any anxiety and depression symptoms. (See Table 3).

**Table 3:** Level of psychological factors among study population on antenatal care follow-up at public health institution in Debre Berhan, April 2018(*n* = 380)

Variables		Frequency	Percentage
Social support status	Poor support	43	11.3
	Moderate support	171	45.0
	Strong support	166	43.7
Depression and anxiety	Normal	212	55.8
	Mild	114	30.0
	Moderate	48	12.6
	Severe	6	1.6
Husband support	Poor support	99	26.1
	Moderate support	193	50.8
	Strong support	88	23.2
Practical support from family	Yes	244	64.2
	No	136	35.8
Physical/emotionally abused by a partner	Yes	14	3.7
	No	366	96.3

### The prevalence of risky alcohol use behavior and its associated factors

**Fig. 1:**
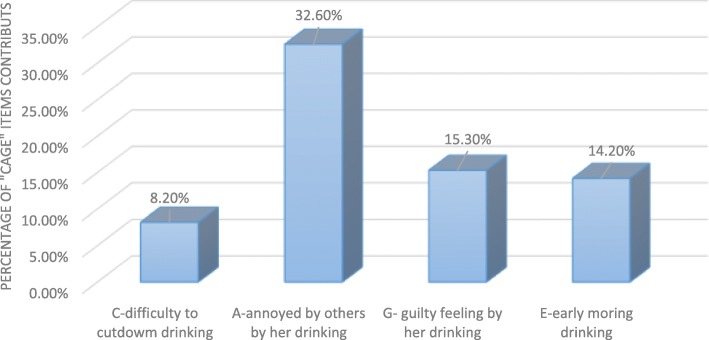
Specific CAGE questionnaire items contribute to the overall prevalence of risky alcohol use during pregnancy

The prevalence of risky alcohol use behavior during pregnancy was screened by using the CAGE questionnaire, and mothers who reported two or more yes were considered as having risky alcohol use behavior. The overall prevalence of risky alcohol use was 16.1% (95% CI: 12.1, 19.7) (Fig. 1).

During Bivariate analysis, twelve variables were significantly associated with the outcome variable with a *p*-value of less than 0.2, and these variable were exported to multivariable binary logistic regression. Among them, only four predictor variables were significantly associated with the outcome variables i.e. risky alcohol use behavior at p-value < 0.05.

Having Poor social support were associated with risky alcohol use behavior during pregnancy. The odds of having risky alcohol use behavior were four times higher in odds among mothers who had poor social support as compared with who had strong social support (AOR = 4.06, 95% CI = 1.56,10.5).

Having diagnosed with family psychiatric history was higher in odds to use alcohol in a risky way. The odds of having risky alcohol use behavior during pregnancy were two times higher among mothers who had diagnosed family psychiatric history as compared with those who had no family psychiatric history (AOR = 2.20, 95% CI = 1.03,5.41). In a similar dimension, being suffered moderate to severe anxiety and depression during pregnancy was also significantly associated with risky alcohol use behavior during pregnancy (AOR = 1.91, 95% CI = 1.01, 4.50). Among obstetric factors, having a history of abortion in previous pregnancies were associated with risky alcohol use behavior as compared with those mother without a history of abortion; AOR = 1.94, 95% CI = 1.23, 4.41 (see Table 4).

**Table 4:** Bivariate and Multivariate analysis of factors associated with risky alcohol use behavior among mothers who had antenatal care follow up at perinatal care clinics, Debre Berhan, Ethiopia, 2018

Variables		Risky alcohol use		COR (95% CI)	AOR(95% CI)
		Yes	No		
Age category	18–24 (youth)	19	51	2.38 (1.28,4.42)	1.03 (0.40,2.64)
	25–45(young adult)	42	268	1.00	1.00
Average monthly income	≤ 1200 ETB	28	69	3.09 (1.64,5.82)	1.29 (0.56,2.98)
	1201–2500	12	90	1.02 (0.48,2.16)	0.85 (0.35,2.06)
	> 2500	21	160	1.00	1.00
Husband support	Poor	8	14	2.90 (1.35,6.24)	1.65 (0.63,4.31)
	Moderate	20	91	0.86 (0.391.86)	0.77 (0.31,1.96)
	Strong	20	155	1.00	1.00
Social support	Poor support	17	20	4.77 (2.21,10.30	4.06 (1.56,10.5)^*^
	Moderate support	22	137	1.19 (0.63,2.25)	0.97 (0.46,2.07)
	Strong support	28	126	1.00	1.00
Family size excluding self and husband	1 to 2	28	75	2.35 (1.06,5.21)	0.83 (0.25,2.71)
	3 to 4	23	181	0.80 (0.36,1.77)	0.71 (0.16,3.25)
	≥5	10	61	1.00	1.00
Gravidity	First	32	76	3.49 (1.84,6.61)	1.54 (0.40,5.90)
	Second	11	94	0.97 (0.44,2.14)	0.75 (0.28,2.0)
	third	18	149	1.00	1.00
Previous diagnosed psychiatric history	Yes	8	15	3.06 (1.24,7.57)	2.33 (0.76,7.2)
	No	53	304	1.00	1.00
Family diagnosed psychiatric history	Yes	14	31	2.78 (1.37,5.59)	2.20 (1.03,5.41)*
	No	47	288	1.00	1.00
Depression and anxiety	Normal	25	187	1.00	1.00
	Mild	21	93	1.69 (1.00,3.18)	0.88 (0.40,1.92)
	Moderate to severe	15	39	2.88 (1.39,5.95)	1.91 (1.01,4.50)*
History of abortion	Yes	23	45	3.69 (2.01,6.76)	1.94 (1.23,4.41)*
	No	38	274	1.00	1.00
History of still birth	Yes	12	26	2.76 (1.31,5.83)	1.76 (0.65,4.78)
	No	49	293	1.00	1.00
Previous pregnancy complication	Yes	20	48	2.75 (1.49,5.10)	1.48 (0.67,3.30)
	No	41	271	1.00	1.00

Key: *P* value of Hosmer Lemeshow = 0.54 and * = significant variables with *p* value = < 0.05

## Discussion

### Prevalence of risky alcohol use behavior

The current study attempted to determine the prevalence of risky alcohol use behavior during pregnancy and its associated factor. The overall prevalence of risky alcohol use behavior in the current study was 16.1% (95% CI: 12.1, 19.7).

The current study finding shows that the prevalence of risky alcohol use behavior was consistent with studies done in the world health organization’s survey report among Africa countries’ i.e. Cameron (12.6%), Uganda (20.5%), Namibia (14.2%), Sierra Leone (14.8%) [15], Ghana 20.4% [16] and Korea 16.4% [17].

The prevalence of risky alcohol use behavior during pregnancy in this study was higher than those studies conducted in equatorial guinea (2.2%), Seychelles (3.4%), Botswana (5.7%), Eastern Mediterranean (0.2%) [13], and united states national survey 7.6% [18], united states 10% [19], South Africa 5.4% [20] and Nigeria [21]. The higher prevalence of risky alcohol use behavior during pregnancy in the current study might be due to the difference in perceived seriousness of alcohol use during pregnancy. And also the studies in African countries described above were used random-effects meta-analysis to determine alcohol use during pregnancy in some WHO Africa regions. The studies done in the United States [18, 19] were used a national survey and the perceived risk of using alcohol during pregnancy might be well understood (better health literacy) in developed countries.

But, the prevalence of risky alcohol use behavior during pregnancy in the current study was lower than the studies done in Ireland 60% [13], Belarus 47% [22], Denmark 46% [20], United Kingdom and Northern Ireland 41% [20], Russian Federation 37% [20], northern Tanzania 34.1% [23] and Bahir Dar; Ethiopia 34% [24]. The lower prevalence in the current study might be due to, the current study focuses on risky alcohol use than any amount of alcohol consumption.

### Associated factors of risky alcohol use behavior

This study result showed that prevalence of risky alcohol use behavior was higher among pregnant women who had a history of abortion in a previous pregnancy, poor social support, diagnosed family psychiatric history and suffered moderate to severe depression and anxiety. The association of having moderate to severe depression and anxiety with problematic alcohol use in the current study was in agreement with the study done in Australia [25]. The association might be due to the fact that individuals who had poor mental health are prone to use alcohol for self-treatment.

Having a history of abortion in a previous pregnancy, having poor social support and having diagnosed family psychiatric history were also associated with the prevalence of risky alcohol use behavior in the current study. The association might be due to women who had pregnancy-related complications and poor social support are at greater risk to use alcohol to suppress stressful feelings.

Having diagnosed with family psychiatric history including depression will also make individuals prone to have poor mental health and concurrently may use alcohol for stress management.

### “CAGE” questionnaire

World health organization report on alcohol drinking during pregnancy recommended that there is no safe amount and time of alcohol drinking, especially for the growing fetus. But, the Current study authors use “CAGE” questionnaire to assess risky alcohol use for both the mother and the growing fetus. The CAGE screening tool was used for this study opposed to alternate single question screening tools due to the authors hypothesize that since local drinks are easily available at the study area in addition to modern drinks pregnant mothers may use alcohol at least once in the current pregnancy by ignoring an unfavorable outcome. And the authors aimed to determine the proportions of risky alcohol drinkers to herself and the fetus than using the single question asking about alcohol use during pregnancy. But, the authors had no additional reason for using CAGE screening tool than other standardized tools like T-ACE and AUDIT.

## Limitation of the study

This study did not assess alcohol use biomarker tests. Since local drinks alcohol by volume and amount of drinks in ounce are difficult to determine; the number of standard drinks consumed by pregnant women per day was not assessed. Social desirability bias may underestimate the prevalence of risky alcohol use.

## Conclusions

According to world health organization recommendation, it is expected to no pregnant women will drink alcohol in any amount. But, in the current study, a significant proportion of mothers drink alcohol in a risky way (for both the mother and the growing fetus). Having known family psychiatric illness, having a history of abortion, having moderate to severe depression and anxiety, having poor social support were found to be significantly associated factors with risky alcohol use behavior during pregnancy. Therefore, the current study authors failed to accept both the null hypothesizes.

## Recommendations

It will be better if screening of little alcohol use (risky for the fetus) and risky use of alcohol (risky for both the fetus and the mother), and providing health education about the effect of alcohol use to the fetus and to her for all pregnant women at antenatal care follow up unit.

## Data Availability

The datasets used and/or analyzed during the current study are available from the corresponding author on reasonable request.

## Abbreviations

ANC: Ante-Natal Care
AOR: Adjusted odds ration
CAGE: C-cut down, A-annoyed, G-Guilty, and E-eye opener
COR: Crude odds ratio
FAS (D): Fetal Alcohol Syndrome (disorder)
SPSS: Statistical Package for Social Sciences
WHO: World Health Organization
